# Correlation between Breast Arterial Calcification and the 10-year fatal cardiovascular risk by means of the SCORE Risk System

**DOI:** 10.12669/pjms.346.16031

**Published:** 2018

**Authors:** Ahmet Yildiz, Seyma Yildiz, Huseyin Toprak, Cuneyt Kocas

**Affiliations:** 1 *Ahmet Yildiz, Istanbul University, Institute of Cardiology, Department of Cardiology, Istanbul, Turkey*; 2 *Seyma Yildiz, MD. Bezmialem Vakif University, Department of Radiology, Istanbul, Turkey*; 3 *Huseyin Toprak, MD. Bezmialem Vakif University, Department of Radiology, Istanbul, Turkey*; 4 *Cuneyt Kocas, MD. Istanbul University, Institute of Cardiology, Department of Cardiology, Istanbul, Turkey*

**Keywords:** Breast arterial calcification, Mammography, SCORE risk system

## Abstract

**Objectives:**

The aim of this study was to investigate the relationship between Breast Arterial Calcification (BAC) on mammography and the 10-year fatal Cardiovascular Disease (CVD) risk by using SCORE risk system.

**Methods:**

The study was conducted from September 2013 to July 2014. A total of 66 women with BAC and 66 age-matched controls without BAC were analyzed. The groups were compared with respect to demographics, clinical, reproductive, laboratory parameters, and 10-year fatal CVD risk.

**Results:**

The mean ages of the women in the study was 54.0 years (40-85 years). Hypertension, systolic blood pressure, levels of serum total cholesterol and the calculated SCORE risk were higher in the BAC (+) group than in the BAC (-) group (p=0.04, p=0.031, p=0.046, and p=0.038 respectively). Multivariate analysis showed that none of them was independent factor of BAC on mammograms, only the 10-year fatal CVD risk was close to being statistically significant (OR:1.17, CI:0.98-1.38, p=0.06).

**Conclusion:**

BAC on mammography was found to be related to the 10-year fatal CVD risk as calculated by the SCORE risk score system. Additional large-scale prospective studies are required to further assess whether BAC can be considered a useful screening tool for CVD risk prediction in women who screened for breast cancer by mammography.

## INTRODUCTION

Heart diseases and cancer are the leading causes of death among women in the US, and breast cancer (BC) is the most common malignancy in women.^1^ According to the current clinical practice guidelines of the US Preventive Services Task Force, mammography is recommended as a screening test for the early diagnosis of BC for all women older than 40 years of age.^2^ Breast Arterial Calcification (BAC) appearing as linear calcium deposition on arterial walls is a frequent benign finding on mammography and is unrelated to malignancy.^3^ Several studies have shown that BAC is associated with Diabetes Mellitus (DM), hypertension, Metabolic Syndrome (MS), Carotid Intima Media Thickness (C-IMT), and Cardiovascular Disease (CVD).^4^,^5^ Women with coronary artery disease have worse prognosis compared with men, and its prevalence increases with age, especially for those older than 50 years.^6^,^7^ Clinical risk scores are useful tools to facilitate risk estimation in healthy people. SCORE risk estimation system is recommended to calculate 10-year fatal risk of CVD.^8^,^9^ The SCORE system includes age, gender, blood pressure, current smoking status, and total cholesterol levels. The Framingham 10-year Coronary Heart Disease (CHD) risk score is a strong predictor of Coronary Artery Disease (CAD), and its relationship between BAC has been investigated^5^, but to date the estimated 10-year fatal risk of CVD in women with BAC has not been validated by using the SCORE risk system. Therefore, the aims of this study was to investigate the relationship between BAC on mammography and the 10-year fatal CVD risk by using SCORE risk system.

## METHODS

From September 2013 to July 2014, a total of 2,780 women older than 40 years of age underwent screening of digital mammography for BC. Excluded from the study were patients who had prior breast surgery or presence of trauma, presence of CVD, or cerebral vascular disease, as well as patients with concomitant inflammatory diseases, or a major illness such as cancer, liver disease, and renal insufficiency. Among these subjects, 66 women with BACs (the BAC positive group) and 66 age-matched controls without BACs (the BAC negative group) were included in the analysis (range 40 to 85 years, mean age 54.0 years). The baseline questionnaire collected demographic information such as name, date of birth, and contact information. Risk factors for CHD also were assessed, including the presence of hypertension, DM, hypercholesterolemia, and smoking history. Other medical and reproductive history (number of childbirths, age at menopause) plus detailed physical examination were recorded in all cases. Fasting glucose, total cholesterol, High-Density Lipoprotein (HDL) cholesterol, Low-Density Lipoprotein (LDL) cholesterol, and triglyceride levels, were measured. This study was reviewed and approved by the ethics committee, all eligible individuals were given an explanation of the research by the investigator, and informed consent was obtained from all participants.

### Baseline definitions and measurements

Hypertension was defined as diastolic blood pressure ≥90 mmHg or systolic blood pressure ≥140 mmHg or by a subject’s use of an antihypertensive drug. The diagnosis of DM was made if the fasting plasma glucose concentration was ≥126 mg/dl on two different measurements, or if the patient was on treatment with insulin or oral glucose-lowering agent(s). Body mass index (kg/m2) was calculated by dividing patient weight (kg) by height (m^2^). Waist circumference measurements were taken at the end of normal expiration and to the nearest 0.1cm, measuring from the narrowest point between the lower borders of the rib cage and the iliac crest.

### Mammography technique and breast arterial calcification

**Fig.1:**
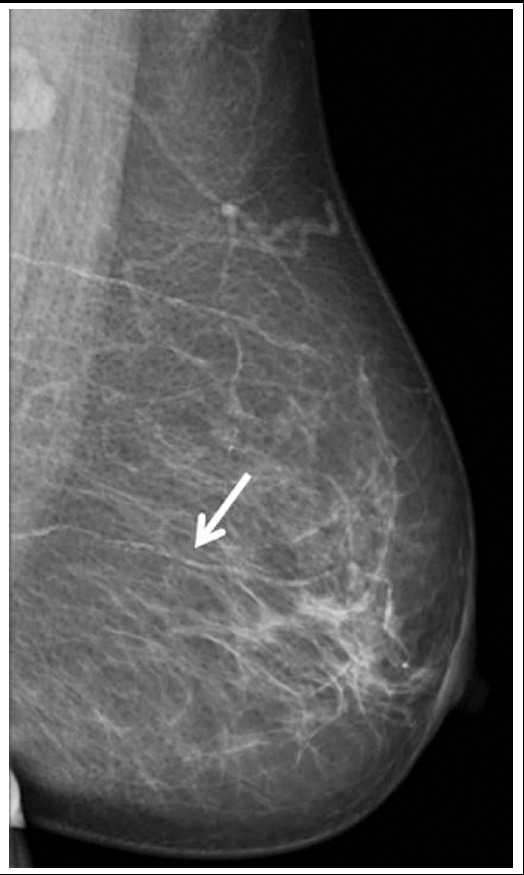
Left mediolateral oblique mammogram showing arterial wall calcifications in a 58-year-old woman (arrows).

Mammograms were analyzed by single experienced radiologist according to the breast reporting system recommended by the American College of Radiology^10^, who was blinded to the results of the questionnaire. Full field digital mammographic examination of the participants was performed in the bilateral standard, mediolateral oblique, and craniocaudal positions (Mammomat Inspiration, Siemens, Erlangen, Germany). BAC was characterized by the presence of two linear calcium depositions along the periphery of the configuration of tapered structures typical of arteries and distinct from breast ducts (Fig.1).

### The 10-year risk of fatal CVD

This study used the SCORE system (www.HeartScore.org) for participants, aged 40 to 85 years, in order to estimates the participants’ 10-year risk of a first fatal atherosclerotic event, whether heart attack, stroke, aneurysm of the aorta, or other cause. The 10-year risk of fatal CVD risk was based on the following risk factors: age, gender, smoking status, systolic blood pressure, and total cholesterol.

### Statistical Analysis

Statistical analysis was performed using the SPSS for Windows software, version 15 (Chicago, Illinois). Continuous variables are expressed as mean ± standard deviation, whereas categorical variables are displayed as numbers and percentages. Student t-test and nonparametric Mann-Whitney U test were used to determine the differences between mean values for normally and non-normally distributed variables, respectively. Categorical variables were reported as percentages and were analyzed by either the chi-square or the Fisher exact test, as appropriate. Multiple logistic regression analysis was used to identify the factors related to breast arterial calcification. All tests were 2 sided, and a significance level of 5% was used.

## RESULTS

The demographic, clinical, and reproductive characteristics parameters of the participants are shown in Table-I. Hypertension, DM, dyslipidemia, number of childbirths, menopausal age, and number of postmenopausal patients, were higher in the BAC (+) group than in the BAC (-) group, but the differences were not statistically significant except for hypertension (p=0.04). No significant differences arose in baseline BMI or waist circumference between the BACs positive group and the BACs negative group. The number of current smokers was higher in the BAC (-) group, but this was not statistically significant (30.3% vs. 16.7%, p=0.06). The laboratory finding and calculated SCORE (%) 10-year risk of fatal CVD- risk scores of the BAC (+) and BAC (-) groups are presented in Table-II. In the BAC (+) group, systolic blood pressure (139.5±16.9 vs. 131.0±19.1, p=0.031) and levels of serum total cholesterol (239.7±32.5 vs. 224.1±29.5, p=0.046) were also higher than in the BAC (-) group. HDL levels of the BAC (+) group were lower than in the BAC (-) group with a difference that neared statistical significance (51.9±14.5 vs. 62.6±17.1, p=0.05). The calculated SCORE (%) was significantly higher in the BAC (+) group than in the BAC (-) group (2.64±2.45 vs. 2.02±1.92, p=0.038).

**Table-I:** The demographic, clinical and reproductive characteristics parameters of the BAC (+) and BAC (−) groups.

	BAC (+) (n = 66)	BAC (-) (n = 66)	P value
Age (years)	54.09±11	54.09±10.8	1
Body mass index (kg/m^2^)	30.2±5.8	28.8±4.9	0.48
Waist circumference (cm)	87.9±13.6	86.1±11.4	0.99
Cigarette smoking (%)	11(16.7)	20(30.3)	0.06
Hypertension (%)	29(43.9)	17(25.8)	0.04
Diabetes mellitus (%)	11(16.7)	5(7.6)	0.13
Dyslipidemia (%)	20(30.3)	18(27.3)	0.71
Number of infant deliveries (n)	4.1±1.3	3.7±1.2	0.54
Age at menopause (years)	49.2±10.9	50.8±12.5	0.87
Number of postmenopausal patient (%)	55(83.3)	51(77.3)	0.21

BAC, breast arterial calcification.

**Table-II:** The laboratory parameters and 10 year fatal CVD risk of the BAC (+) and BAC (−) groups.

	BAC (+) (n = 66)	BAC (-) (n = 66)	P value
Systolic blood pressure (mmHg)	139.5±16.9	131.0±19.1	0.031
LDL-cholesterol (mmol/L)	136.8±32.3	131.5±30.4	0.50
HDL-cholesterol (mmol/L)	51.9±14.5	62.6±17.1	0.05
Triglycerides (mmol/L)	156.9±80.1	140.4±69.5	0.42
Total cholesterol (mmol/L)	239.7±32.5	224.1±29.5	0.046
Fasting glucose (mg/dL)	117.5±44.7	109.4±38.4	0.33
The 10 year fatal CVD risk (SCORE) (%)	2.64±2.45	2.02±1.92	0.038

BAC, breast arterial calcification; LDL, low-density lipoprotein; HDL, high-density lipoprotein; CVD, cardiovascular disease.

### Multivariate Analysis

The multivariate analysis was performed to determine the relationship of BAC and 10-year fatal CVD risk calculated by the SCORE risk score system. While presence of BAC on mammograms was significantly related to 10-year fatal CVD risk as calculated by univariate analysis, it was not an independent factor in multivariate analysis although it was close to being statistically significant (OR:1.17, CI:0.98-1.38, p=0.06) (Table-III).

**Table-III:** Results of multivariate analysis for the presence of BAC.

	Odds Ratio	95% CI	P value
Cigarette smoking	1.5	0.5-2.5	0.14
Hypertension	1.9	0.14-6.7	0.61
Diabetes mellitus	0.95	0.61-2.5	0.16
Total cholesterol	1.2	0.99-1.06	0.12
Systolic blood pressure	1.0	0.93-1.08	0.80
HDL-cholesterol	0.95	0.88-1.01	0.13
The 10 year fatal CVD risk (SCORE)	1.17	0.98-1.38	0.06

BAC, breast arterial calcification; HDL, high-density lipoprotein; CI, confidence interval; CVD, cardiovascular disease.

## DISCUSSION

In this study, and for the first time in the literature, we observed that BAC is associated with an increased prevalence of a 10-year fatal risk of CVD as calculated with the SCORE risk system. The US Preventive Services Task Force’s current clinical practice guidelines recommend mammography for all women above the ages of 40 as a screening test for the early diagnosis of BC.^2^ Breast arterial calcification is a benign finding typically seen on mammography and is identified as medial calcific sclerosis of small- to medium-sized muscular arteries of the breast.^11^ In our study, prevalence of BAC detected by mammography was 13.6 % among 2.780 women, similar to what has been reported in previously published studies, which varies from 1% to 49%.^4^ A relationship between BAC and reproductive factors such as duration of breast feeding, number of infant deliveries, age of menopause, and duration of menopause has been investigated in several studies.^11^-^13^ Additionally, presence of BAC on mammography was shown to be associated with age, hypertension, DM, presence of CAD, peripheral arterial disease, presence of coronary artery calcification, C-IMT and metabolic syndrome.^4^,^5^,^14^-^17^

In our study, the numbers of infant deliveries, number of postmenopausal patients, DM, and hypertension increased with BAC, but they were not independent predictors of BAC. CVD is the leading cause of death worldwide; it is on the rise and having become a true pandemic. The most important reason is, the increasing prevalence of cardiovascular risk factors, but 20% of all coronary events occur in the absence of major cardiovascular risk factors, and 60% of events are experienced by low-to-intermediate risk patients.^18^-^20^ For that reason, early detection and estimation of cardiovascular risk is very important. Nowadays there is great interest in developing new methods, including novel serum biomarkers, noninvasive imaging modalities, and clinical risk scores to better identify patients who may be appropriate candidates for more aggressive cardiovascular primary prevention.^21^,^22^ There are many well-known clinical risk scores to predict the risk profile of an individuals. The most known and used risk scores; are the Framingham, SCORE, PROCAM, and QRISK WHO / ISH, as well as the Reynolds Risk Score, which is a risk calculation system consisting of an organized table to determine the absolute risk of cardiovascular events.^8^ In European countries, the SCORE risk score is used for estimating 10-year fatal cardiovascular risk based on levels of systolic blood pressure, smoking status, gender, total cholesterol, and age. According to percentage of risk, people are classified into four groups (< 1% is considered low risk, 1-5% is moderate risk, 5-10% high risk, and > 10% very high risk). In our study we found that the SCORE risk score was higher in the BAC (+) group than in the BAC (-) group, whereas for both groups of participants, the mean SCORE risk scores were in the moderate risk group (1-5%). While presence of BAC was significantly related to 10-year fatal CVD risk by univariate analysis, it was not an independent factor in multivariate analysis, but it was nearly statistically significant (OR:1.17, CI:0.98-1.38, p=0.06). Bae et al.^5^ suggested that 10-year cardiovascular risk calculated based on the Framingham Score was significantly higher in the BAC (+) group than that of the BAC negative group (p=0.007). Women are more likely to present atypical symptoms, so it is important that cardiovascular risk screening and prevention be maximized. Especially for patients in the moderate risk group, additional new risk predictors should be considered. According to our results, for patients in the moderate risk group, BAC positiveness may be used as a new risk predictor in terms of CVD. Smoking is associated with increased risk of all types of CHD, according to estimations from SCORE, 10-year fatal cardiovascular risk is approximately doubled in smokers.^23^ The inverse relationship between smoking and BAC as shown in the literature, was confirmed in the present study.^4^,^24^ This inverse relationship may be explained by the lack of inflammatory reactions on the pathogenesis of BAC. Our study revealed that many of the risk factors of CHD such as hypertension, DM, hyperlipidemia, and obesity are not related to BAC. Our study showed that BAC is not associated with a specific disease but may instead be related to the summation of different risk factors.

### Limitations of the study

Because of the relatively small sample size, the results may not be representative of the general population. Also, although individuals with known CAD were excluded from the study, asymptomatic patients with coronary artery disease could not be ruled out in study groups, because we did not perform coronary angiography in any of the groups.

## CONCLUSION

BAC on mammography was found to be related to 10-year fatal CVD risk by the SCORE risk score system. Since a relatively small number of patients were included in this study, additional large-scale prospective studies are required to further assess whether BAC can be considered a useful screening tool for CVD risk prediction in women who screen for breast cancer by mammography.

### Author’s contribution

**SY, AY and CK:** Designed the study, performed the statistical analysis of results and wrote the manuscript.

**HT:** Collected the patient data and contributed to the discussion section.
